# Exoskeleton-Robot Assisted Therapy in Stroke Patients: A Lesion Mapping Study

**DOI:** 10.3389/fninf.2018.00044

**Published:** 2018-07-17

**Authors:** Antonio Cerasa, Loris Pignolo, Vera Gramigna, Sebastiano Serra, Giuseppe Olivadese, Federico Rocca, Paolo Perrotta, Giuliano Dolce, Aldo Quattrone, Paolo Tonin

**Affiliations:** ^1^S. Anna Institute and Research in Advanced Neurorehabilitation (RAN) Crotone, Crotone, Italy; ^2^Neuroimaging Unit, IBFM-CNR, Catanzaro, Italy; ^3^Neuroscience Research Centre, University Magna Græcia, Catanzaro, Italy

**Keywords:** stroke, neurorehabilitation, exoskeleton, upper limb, voxel-based lesion symptom mapping

## Abstract

**Background**: Technology-supported rehabilitation is emerging as a solution to support therapists in providing a high-intensity, repetitive and task-specific treatment, aimed at improving stroke recovery. End-effector robotic devices are known to positively affect the recovery of arm functions, however there is a lack of evidence regarding exoskeletons. This paper evaluates the impact of cerebral lesion load on the response to a validated robotic-assisted rehabilitation protocol.

**Methods**: Fourteen hemiparetic patients were assessed in a within-subject design (age 66.9 ± 11.3 years; 10 men and 4 women). Patients, in post-acute phase, underwent 7 weeks of bilateral arm training assisted by an exoskeleton robot combined with a conventional treatment (consisting of simple physical activity together with occupational therapy). Clinical and neuroimaging evaluations were performed immediately before and after rehabilitation treatments. Fugl-Meyer (FM) and Motricity Index (MI) were selected to measure primary outcomes, i.e., motor function and strength. Functional independance measure (FIM) and Barthel Index were selected to measure secondary outcomes, i.e., daily living activities. Voxel-based lesion symptom mapping (VLSM) was used to determine the degree of cerebral lesions associated with motor recovery.

**Results**: Robot-assisted rehabilitation was effective in improving upper limb motor function recovery, considering both primary and secondary outcomes. VLSM detected that lesion load in the superior region of the corona radiata, internal capsule and putamen were significantly associated with recovery of the upper limb as defined by the FM scores (*p*-level < 0.01).

**Conclusions**: The probability of functional recovery from stroke by means of exoskeleton robotic rehabilitation relies on the integrity of specific subcortical regions involved in the primary motor pathway. This is consistent with previous evidence obtained with conventional neurorehabilitation approaches.

## Introduction

Several systematic and meta-analytic reviews have confirmed that robotic-assisted devices elicit robust motor recovery in patients with stroke, mainly in relation to the upper limb intervention (Masiero et al., 2007; Bertani et al., 2017; Lo et al., 2017). Early research on robotic therapy for the upper limb was based on end-effector robots, which hold the patient’s hand or forearm at one point and generate forces at the interface. Recently this field of study has shifted towards an exoskeleton device, which overcomes many of the inherent limitations of end-effector robots (Pignolo, 2009; Lo and Xie, 2012). Compared to conventional therapy, exoskeletons have the potential to provide intensive rehabilitation consistently for a longer duration and irrespective of the skills and fatigue level of the therapist (Huang and Krakauer, 2009; Lo and Xie, 2012).

The Automatic Recovery Arm Motility Integrated System (ARAMIS) is a concept robot and prototype for the neurorehabilitation of the paretic upper limb developed at the Institute S. Anna—Crotone, Italy. ARAMIS was designed with two computer-controlled, symmetric and interacting exoskeletons, which compensate for the inadequate strength and accuracy of the paretic arm movements and the effect of gravity during rehabilitation. The basic idea is to exploit proprioceptive inputs using passive, repetitive, interactive, high-intensive bilateral movement training, which has been demonstrated to enhance motor recovery in stroke patients (Stinear et al., 2008; Choo et al., 2015; Saleh et al., 2017; Gandolfi et al., 2018). This device has been widely validated (Colizzi et al., 2009; Dolce et al., 2009; Pignolo et al., 2012) with respect to conventional neurorehabilitation approaches, demonstrating high degree of upper limb recovery as assessed by the Fugl-Meyer (FM) scale (Fugl-Meyer et al., 1975). The FM is a performance-based impairment index designed to assess motor functioning, balance, sensation and joint functioning in patients with post-stroke hemiplegia. Overall, FM together with the Modified Ashworth Scale (MAS) and functional independance measure (FIM) (Keith et al., 1987), are the most reliable clinical scales employed to unravel motor recovering after robotic-treatment in stroke patients (Bertani et al., 2017).

Despite this large amount of evidence confirming the effectiveness and robustness of robotic-assisted rehabilitation in promoting motor recovery the underlying pathophysiology is still unclear. In fact, some biomarkers have been effectively demonstrated to predict therapeutic response or recovery following stroke (Burke Quinlan et al., 2015). Generally, research has focused on the neural substrate of motor recovery obtained with a conventional neurorehabilitation approach (Shelton and Reding, 2001; Murphy and Corbett, 2009; Carrera and Tononi, 2014; Choo et al., 2015; Lee et al., 2017; Siegel et al., 2018), whereas little attention has been paid to robotic-assisted therapy with exoskeleton devices (Formaggio et al., 2013; Fan et al., 2016; Gandolfi et al., 2018). Overall, preservation of the corticospinal tract is considered as a hallmark for good recovery of impaired motor function in patients with brain injury (Hendricks et al., 2002; Swayne et al., 2008; Stinear, 2010). Within this pathway there are several critical hubs that have been associated with functional recovery after conventional therapy. Mounting evidence from functional magnetic resonance imaging (fMRI), diffusion tensor imaging (DTI) as well as resting-state functional connectivity studies have demonstrated that the increased activity in ipsilateral primary motor cortex and the morphological integrity of the posterior limb of the capsula interna predict the positive clinical outcome (Shelton and Reding, 2001; Stinear et al., 2012; Stinear and Ward, 2013; Favre et al., 2014; Rehme et al., 2015). Moreover, lesions in the globus pallidus, and putamen (together with corona radiata, internal capsule) were also associated with poor recovery (Lee et al., 2017).

This study, thus, assesses the effects of lesion location on the response to rehabilitation training obtained with an exoskeleton robot device. The aim is to expand knowledge of the neural basis of stroke rehabilitation and the prognosis of upper limb disorders.

## Materials and Methods

### Subjects

**Figure 1:**
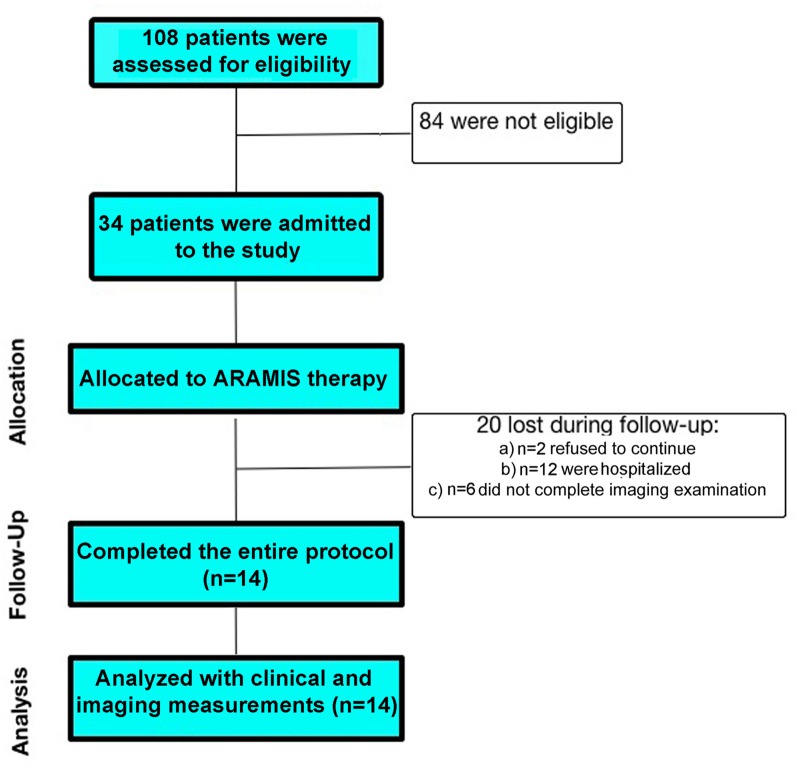
Flow diagram of participant recruitment and participation in the study: stroke patients participated in an individualized robotic-assisted neurorehabilitation program (ARAMIS system).

We enrolled patients who met the criteria for a first attack of sub-cortical ischemic stroke recruited at the Sant’Anna Rehabilitation Center. From an initial cohort of 108 subacute hemiplegic patients, we enrolled only those who fulfilled the following criteria: (i) unilateral stroke, (ii) ability to follow verbal instructions, and (iii) right-handed patients. Exclusion criteria were (1) bilateral impairment; severe sensory deficits in the paretic upper limb; (2) pregnancy, epilepsy, aphasia, cognitive impairment (Mini Mental State Evaluation, MMSE < 24) or behavioral dysfunction that would influence the patient’s ability to comprehend or participate in the treatment; (3) botulinum toxin injections or other medication influencing the function of the upper-limb; (4) inability to provide informed consent and (5) and/or pacemakers or other metallic implants incompatible with the 3T MRI scanner. From the initial cohort, 34 patients were selected for the rehabilitation program (Figure 1).

All the participants gave written informed consent. The study was approved by the Ethical Committee of the University “Magna Graecia” of Catanzaro, according to the Helsinki Declaration.

All patients completed an extensive series of clinical tests that were administered by an experienced physician who was blind to any other result. The degree of disability during daily living activities was assessed with the Barthel Index (Collin et al., 1988) and the motor strength of the upper-limb was assessed with the Motricity Index (MI; Collin and Wade, 1990). Patients’ synergistic motor control of the paretic arm was assessed with the upper extremity (UE) section of the FM (FM-UE; Lindmark, 1988). Further measures included the FIM and the Trunk Control Test (TCT; Franchignoni et al., 1997).

### ARAMIS Hard/Software Structure

**Figure 2:**
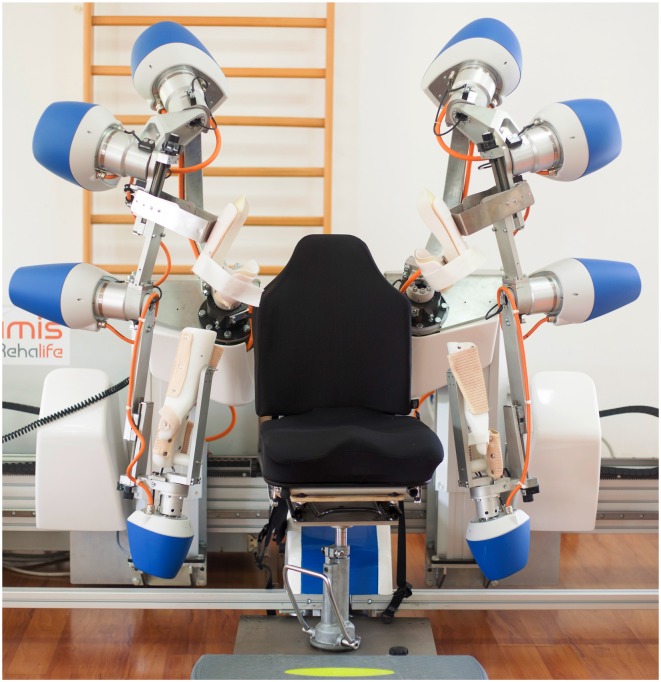
The robotic-assisted device called Automatic Recovery Arm Motility Integrated System (ARAMIS).

The robotic-assisted rehabilitation performed through the ARAMIS device has been described elsewhere (Colizzi et al., 2009; Dolce et al., 2009; Pignolo et al., 2012). The ARAMIS framework is a fully integrated set of software that enables the therapist to program and manage the rehabilitation procedures. Briefly, the robotic platform includes two fully-motorized 6 DOF symmetric exoskeletons (Figure 2). Kinematic and dynamic data are jointly continuously acquired and stored by the control system, which evaluates the weight torque and compensates for it by controlling each upper limb posture and the strength delivered by the patient to the exoskeleton. Movements are, therefore, supported by a drive motor, which adjusts its strength on a step-by-step basis.

Each exoskeleton can record (motion capture) the movements of the unaffected arm and the patient is requested to replicate each single movement by the paretic arm in synchronous or asynchronous modalities depending on the exercise typology or training program, with continuous compensation for the paretic arm’s inadequate strength and accuracy. ARAMIS-assisted rehabilitation is possible in three different modalities: (1) asynchronous: the patient wears both exoskeletons and uses the unaffected arm to perform pre-programmed exercises that are replicated by the paretic arm supported by its own exoskeleton; (2) synchronous: the unaffected arm paces the movements to be replicated synchronously and with the same physical characteristics (such as strength, acceleration, range, and speed) by the exoskeleton hosting the paretic arm; (3) active-assisted: when the patient is not able to carry out a movement, the robot supports the arm strength against gravity, thus replicating movements executed by the unaffected arm.

### Design and Procedure

**Figure 3:**
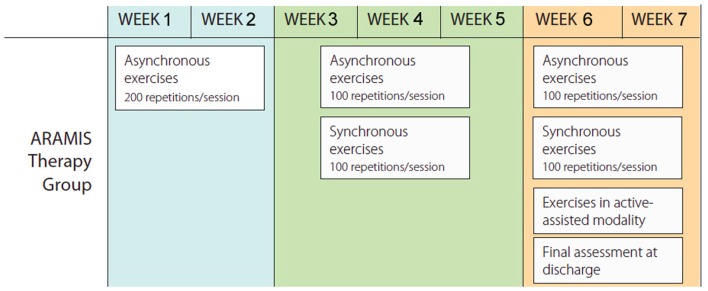
Therapy protocol with ARAMIS.

We used a within-subject design divided into four main stages. The first stage was based on the recruitment of the patients (see inclusion criteria reported above). Physiotherapists as well as data entry assistants were blinded to all phases of the study. In the second stage, the eligible stroke patients underwent an MRI examination at baseline (T0). In the third stage, participants underwent a rehabilitation program consisting of a validated protocol of exoskeleton-robot assisted activities (Colizzi et al., 2009; Dolce et al., 2009; Pignolo et al., 2012) combined with an additional 4–5 h per week of conventional therapy (Aisen et al., 1997; Volpe et al., 2000), in agreement with Italian norms on the treatment of stroke patients. The conventional activities were carried out by an (blinded) expert therapist and consisted of occupational therapy exercises together with passive/active mobilization of upper and lower limbs, trunk control, standing and ambulation. The robot-assisted rehabilitation programs are summarized in Figure 3 (Colizzi et al., 2009; Dolce et al., 2009; Pignolo et al., 2012). The ARAMIS protocol for rehabilitation included 60-min sessions daily over periods not exceeding 7 weeks. Both single and multiple movements were scheduled. In the first 2–3 weeks of treatment, all subjects performed a series of asynchronous exercises, where the paretic arms repeated each of the exercises 20 times for a total of 200 repetitions per session (Table 1). In the following 2–3 weeks, the asynchronous exercises were gradually reduced to 100 per session and replaced by synchronous exercises (100/session), with the total number remaining unchanged. The rehabilitation sessions in the active-assisted modality began following an adequate motor recruitment of the upper limb where necessary as stipulated in the FM-UE scale modified by Lindmark and Hamrin (1988; total score >70), which continued to the end of scheduled treatment (Figure 3). All patients started rehabilitation periods at the same time.

**Table 1:** Exercises performed with an exoskeleton robotic device.

	Joint	Movements
Simple exercises	Shoulder	elevation: 30°, 60° and 90°
		abduction-adduction: 30°, 60° and 80°
		circling (circle movement on frontal axis)
		flexion-extension
	Elbow	flexion-extension
	Forearm	pronation-supination
Functional exercises	Shoulder and Forearm	Shoulder elevation 90° + Forearm pronation-supination;
	Shoulder and elbow	Shoulder elevation 90° + Elbow flexion-extension;
	Shoulder, elbow and forearm	Shoulder elevation 90° + Elbow flexion-extension + Forearm pronation-supination;
		Shoulder elevation 90° + 2 Elbow intermediate flexion-extension + Forearm intermediate pronation-supination

Finally, at the end of the 7-week training period, participants were given a blinded motor assessment, using the same protocol as at baseline (T1). Twenty patients were removed from the final analysis: (a) two refused to continue; (b) 12 were hospitalized and (c) six patients were removed because they did not complete imaging evaluation. Thus, 14 stroke patients completed the entire protocol.

### MRI Data Acquisition

All participants underwent the same MRI scanning protocol immediately before and after rehabilitation. MRI and clinical assessments were performed in the same day. Patients were examined using a 3-Tesla GE MR750 scanner (GE Healthcare, Rahway, NJ, USA). The MRI protocol included whole-brain, three-dimensional, T1-weighted (BRAVO), spoiled gradient recall echo (TE/TR = 3.7/9.2 ms, flip angle 12°, voxel size = 1 × 1 × 1 mm^3^), T2-weighted images (TR = 3500 ms, TE = 20/85 ms) and fast fluid-attenuated inversion-recovery (FLAIR) axial images (TR = 9500 ms, TE = 100 ms; matrix size 512 × 512, FOV: 24 cm; 36 slices, 4 mm slices, 0 mm gap).

### Lesion Identification

Lesion locations and size was performed, using MRIcron software (version 12, Rorden and Brett, 2000). For each patient, we manually outlined the lesion area (volume of interest (VOI) images) on each slice of the FLAIR images. All lesions were traced by a trained image analyst and by an experienced clinical neurologist and neuroradiologist, who were blinded to all clinical data. These specialists worked together to develop a consensus regarding the extent of the lesion in each individual. The FLAIR scan and the corresponding lesion maps were coregistered onto the T1-weight template MRI scan from the Montreal Neurological Institute (Brett et al., 2001; Rorden et al., 2007). Finally, the T1 scan and the VOI images were mapped into stereotaxic space, using the normalization algorithm provided in the SPM8^1^ software, which is exceptionally robust to the presence of lesions (Seghier, 2008). The number of MRI voxels involved in each stroke lesion was calculated. The lesion size was also derived from the lesion mask by multiplying the number of voxels with voxel volume (1 mm^3^).

### Voxel-Based Lesion-Symptom Mapping (VLSM)

To visualize the lesion locations and evaluate the overlap of the lesioned voxels across participants, MRIcron software was used. The relationship between neuroanatomical damage and motor recovery was evaluated using a well-known validated method: Voxel-Based Lesion-Symptom Mapping (VLSM; Bates et al., 2003).

The normalized images and lesion masks, along with the measures of motor impairment, were used in the VSLM analysis, implemented in the nonparametric mapping (NPM) software (version 12 December 2012; Chris Rorden, Columbia, SC, USA^2^) included in the MRIcron software suite. In the first analysis, in order to verify the presence of lesion changes between the two time-points (before and after rehabilitation), the nonparametric Liebermeister statistical analysis for binary data was used (Rorden et al., 2007). In addition, taking into account the non-normal distribution of the data to determine which clinical parameter correlated with motor recovery a nonparametric test (using the permuted Brunner-Munzel rank order statistic) for continuous data within the NPM software was considered (Rorden et al., 2007).

Both the Liebermeister statistical analysis and Brunner-Munzel test were conducted assuming an* a priori* minimum lesion density threshold. In fact, only voxels that were lesioned in at least 20% of all patients were included in the analysis. This value is based on a conventional threshold regarding the total incidence of lesions in a voxel regardless of behavioral performance (Rorden et al., 2007), which maximizes statistical power, thus reducing the likelihood of error (i.e., unidentified lesions) (Inoue et al., 2014). The color-coded map derived from the nonparametric Brunner-Munzel test with false-discovery rate (FDR) corrections at *P* ≤ 0.05 was then overlaid onto a standard brain template^3^ and used to determine significant differences between lesioned areas and motor behavioral scores.

### Statistical Analysis

Statistical analyses were performed with Statistica Version 6.0^4^. Assumptions for normality were tested for all continuous variables using the Kolmogorov–Smirnov test. Demographical and clinical variables were normally distributed except for lesion volume. A paired-sample *t*-test (two-sided) was used to verify any statistical significant changes in motor scales before and after robotic-assisted rehabilitation. A correlation between clinical evaluations was performed using nonparametric *r*’ Spearman on motor performance defined as delta scores. In fact, clinical changes associated with different motor neurorehabilitation approaches were calculated as the differential between T1 and T0 (Δ) scores. A two-tailed alpha level of <0.05 was used to define significance.

## Results

Fourteen stroke patients (age: 66.9 ± 11.3; 28% female) completed all phases of rehabilitation protocol and were, finally, included in the imaging analysis. The median lesion volume related to stroke damages was of 44.6 [range: 7–146] cm^3^. The average time period between baseline and re-test evaluation was 44.1 ± 13.9 days. The hemiplegic side was properly disturbed among patients (50% left side). After treatment the vast majority of patients showed evident motor recovery: (A) 68.7% improvement in the FM-UE scale (global score: from 63 ± 21 at baseline to 92 ± 27 after treatment; *t*-value = 6.1; *p* < 0.0001); (B) 94.7% in the MI scale (*t*-value = 6.6; *p* < 0.0001); (C) 65, 6% in the FIM (*t*-value = 6.5; *p* < 0.0001); (D) 174% in the Barthel-Index (*t*-value = 7.6; *p* < 0.0001) and (E) 150% improvement in the TCT scale (*t*-value = 4.4; *p* < 0.009). Motor recovery as assessed by the FM-UE scale, correlated significantly with TCT values (*r* = 0.86; *p-level* < 0.001), whilst the Barthel-Index was related to FIM scores (*r* = 0.66; *p-level* = 0.01).

**Figure 4:**
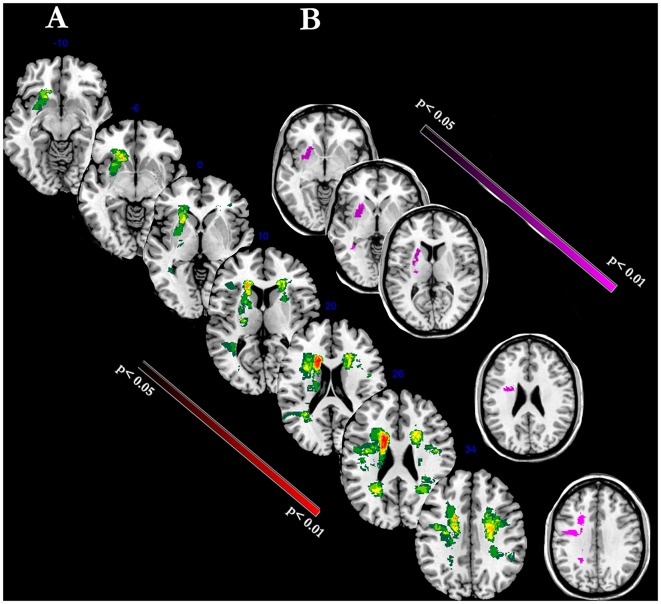
The figure represents voxel-level lesion-mapping analysis performed with voxel-based lesion symptom mapping (VLSM) method implemented in the nonparametric mapping (NPM) software included into the MRIcron software **(A)**. Overlay of FLAIR-dependent MRI lesions detected in all stroke patients (*n* = 14). The color indicates the frequency of overlapping stroke-related lesions (maximal: red blobs). **(B)** Regression Analysis: Voxels within the superior region of the corona radiata, internal capsule and putamen were significantly correlated with the degree of motor recovery as assessed by FM-UE scale (violet blobs; *P* < 0.01).

Figure 4 shows the lesion-mapping analysis. The overlapping lesions of all strokes’ brains mainly included putamen, internal capsule, anterior thalamic radiation, superior/posterior regions of corona radiata (Figure 4A). Lesion mapping analysis was first performed to evaluate the presence of lesion load between the two time-points, without revealing any significant changes. We then performed Voxel-based lesion symptom mapping (VLSM) analysis with a nonparametric approach to evaluate the relationship between each clinical scale and the lesion load and localization. The superior region of the corona radiata, internal capsule and putamen were significantly associated with recovery as assessed by the FM-UE scale (Figure 4B, *p*-level < 0.01). No significant relationship was detected between other clinical scales and lesion mapping. In other words, evidence of motor improvements as defined by MI, FIM, Barthel and TCT scales were not related to lesion load.

## Discussion

Since the beginning of the 21st century, it has been assumed that motor recovery after stroke is widely influenced by lesion location, specially in the subcortical regions which are involved in the primary or secondary motor systems (Shelton and Reding, 2001). Our lesion mapping study confirms that lesion load mainly affecting the primary motor pathway was related to the degree of upper limb motor recovery induced by an exoskeleton robotic-assisted rehabilitation program.

It is worth noting that in the months immediately following a stroke episode, patients can recover between 40%–70% of initial clinical deficits (Ramsey et al., 2017). This behavioral phenomenon is directly dependent on the functional and structural reorganization of specific brain systems (Murphy and Corbett, 2009), which may occur within a few millimeters of lesion borders and may continue over a wide temporal scale (Siegel et al., 2018). Despite the fact that part of this recovery might be spontaneous, it has widely been demonstrated that intensive, repetitive, variable and rewarding motor activities improve functional outcome (Lo et al., 2010; Turner et al., 2013). However, this kind of protocol is time-consuming for conventional manual therapy and given the growing demand due to new cases, alterative automated procedures are needed. As stated by Lo and Xie (2012), robotic-assisted therapy and, in particular, exoskeletons have the potential to provide intensive rehabilitation consistently for a longer duration, thus enabling more frequent treatment and potentially reducing costs. Generally, it has been demonstrated that robot-assisted rehabilitation approaches have a greater effect (although not significant) on motor recovery with respect to conventional therapy (Lo et al., 2010; Bertani et al., 2017). Exoskeleton-robot assisted therapy would seem to increase this effect (Lo and Xie, 2012). This kind of device can accurately control multiple joints at the same time, thus promoting the interaction between the paretic and unaffected upper limbs, which may ultimately aid a patient to produce more realistic task-based exercises (Dolce et al., 2009; Lo and Xie, 2012; Pignolo et al., 2016). However, the neural basis of this clinical effect has been poorly investigated.

Motor recovery in stroke patients has attracted the interest of the neuroimaging community due to the drawbacks of the conventional treatment characterized by simple active/passive strengthening of the affected upper/lower extremities and occupational activity. Generally, motor recovery is typically associated with widespread, synchronous activity modulations in a spatially distributed, highly integrated network encompassing the corticospinal tract (Shelton and Reding, 2001; Lin et al., 2009), The primary motor cortex together with the putamen and capsula interna are all essential components of the brain network involved in restoring of the motor routines necessary for relearning skilled motor behavior (Doyon et al., 2003). In a recent meta-analysis of 24 studies using fMRI of motor tasks, Favre et al. (2014) affirmed that the activity in ipsilateral primary motor cortex is one of the best predictors of the good motor recovery in chronic stroke patients. Similarly, connectivity (i.e., DTI) as well as lesion mapping studies demonstrated that the disruption of the capsula interna, corona radiata, globus pallidus, and putamen predict the functional recovery (Shelton and Reding, 2001; Rehme et al., 2011; Stinear et al., 2012; Stinear and Ward, 2013; Hannanu et al., 2017; Lee et al., 2017). The pathway along the pedunculopontine nucleus, cerebellum, striatum and the motor cortex contributes to motor initiation, modulation of motor rhythm and postural muscle tone during motor behaviors (Takakusaki, 2013). Thus, injuries to the capsula interna, putamen and corona radiata could be considered as a hallmark of motor recovery in stroke patients, especially in the chronic phase (Nudo et al., 1996; Takakusaki, 2013). The few neuroimaging (fMRI and EEG) studies on robotic-assisted devices have described functional compensatory responses in the primary and secondary motor cortices as a function of the increased motor activities related to haptic feedback and bimanual training (Fan et al., 2016; Saleh et al., 2017; Gandolfi et al., 2018).

We believe that this is the first neuroimaging study evaluating how motor recovery obtained with an exoskeleton robot exploits a specific neural substrate. Overall, our lesion map data largely matches previous pathophysiological evidence (Shelton and Reding, 2001; Lo et al., 2010; Turner et al., 2013). However, in contrast to previous studies, one additional finding is worth discussing. In fact, we found that the clinical relevance of neural damage was better express by FM-UE scores, a scale focused on the motor activity of upper limbs. The FM-UE is recognized as the most sensitive clinical scale for detecting the motor recovery of the upper limb functions and is a reliable measure of the neural changes associated with neurorehabilitation (Lo et al., 2010; Hannanu et al., 2017).

Two important limitations need to be discussed. First, the lack of a useful control group for evaluating the impact of robotic-assisted rehabilitation with respect to conventional treatment. Although we are aware of this limitation, it is important to bear in mind that the effectiveness of upper-extremity rehabilitation highlighted by the ARAMIS device with respect to conventional treatment has previously been demonstrated (Colizzi et al., 2009; Dolce et al., 2009; Pignolo et al., 2012). The main target of this study was to investigate how neural damage might affect the motor recovery induced by rehabilitation treatment primarily focused on the employment of an exoskeleton robotic-assisted system rather than evaluating its effectiveness with respect to other approaches. Second, in our study we cannot disentangle the contribution of occupational therapy with respect to robot-assisted rehabilitation since in Italy the guidelines for stroke unit interventions entail combining advanced robotic-assisted rehabilitation with conventional protocol. However, previous studies have already investigated the difference between robotic-assisted therapies in addition to conventional therapy with respect to conventional therapy alone. In particular, Aisen et al. (1997) and Volpe et al. (2000) demonstrated that the outcome of stroke patients increases as a function of intensity and effectiveness of the motor therapy. This, thus, suggests that a combination of rehabilitation approaches is preferable over a single treatment.

Taking into account the fact that our data are similar to findings reported in previous studies regarding the impact of stroke lesions on motor recovery induced by conventional approaches (Turner et al., 2013), all this evidence supports a new hypothesis. In fact, regardless of the neurorehabilitation approaches (conventional vs. robotic-assisted) brain lesions affecting motor recovery are mainly localized in the capsula interna, corona radiata and putamen (Shelton and Reding, 2001). Different kinds of rehabilitation approaches, instead, could be useful to understand which neural networks, subserving functional compensatory responses or strategies, could be exploited to bypass the lesions (Fan et al., 2016; Hannanu et al., 2017). Further fMRI studies investigating the different neural pathways engaged after conventional and robotic-assisted rehabilitation programs are needed. In fact, it has been proposed that the compensatory overactivity of the ipsilateral motor cortex (Favre et al., 2014) is not sufficient to support functional recovery (Ganguly et al., 2009). For this reason, we believe that exoskeleton-robot assisted therapy could exploit different neural networks subserving haptic feedback (Turner et al., 2013; Lo and Xie, 2012).

Overall, as stated by Langhorne et al. (2009), recovery from a stroke event is a complex process that occurs through a potentiation and extension of residual brain areas where lesion location rather lesion volume is more effective in influencing motor recovery. Our data could be useful for monitoring and planning rehabilitation strategies for motor recovery in stroke patients and to highlight the reliability of exoskeleton-robot assisted therapy in addition to other approaches.

## Author Contributions

All authors gave substantial contribution to conception and experimental design, as well as data analysis, interpretation and drafting of the manuscript. The submission of this article has been approved by all authors and by the institution where the work was carried out.

## Conflict of Interest Statement

The authors declare that the research was conducted in the absence of any commercial or financial relationships that could be construed as a potential conflict of interest.

## Footnotes

^1^http://www.fil.ion.ucl.ac.uk/spm/software/spm8

^2^http://www.cabiatl.com/mricro/npm/

^3^http://www.bic.mni.mcgill.ca/ServicesAtlases/Colin27

^4^www.statsoft.com
